# Metabolic and Microbial Mastery: Unveiling the Secrets of Naked Mole-Rat Longevity

**DOI:** 10.1093/geroni/igaf122.1390

**Published:** 2025-12-31

**Authors:** Shelley Buffenstein

**Affiliations:** University of Illinois, Chicago, Chicago, Illinois, United States

## Abstract

Naked mole-rats (Heterocephalus glaber) stand out among mammals for their extraordinary longevity, showing no increase in mortality risk with age and maintaining robust physiological and molecular stability throughout their 40-year lifespan. These subterranean herbivorous rodents have evolved remarkable adaptations for thriving in low-oxygen environments, including the ability to switch from glucose to fructose metabolism, supporting sustained anaerobic activity. Elevated glycogen stores provide a vital energy reserve for anaerobic ATP production, while increased glutathione levels offer a strong defense against oxidative stress, contributing to their exceptional health span and cancer resistance. Metabolomic analyses reveal significant distinctions between naked mole-rats and laboratory mice, notably in their lower plasma levels of methionine pathway amino acids, akin to longevity-associated states such as calorie restriction. Additionally, they exhibit enriched lipid sub-pathways related to membrane composition and sterols, enhancing biological resilience. Elevated concentrations of anti-aging metabolites like α-tocopherol and N8-acetylspermidine further underscore their unique metabolic profile. Unlike mice, naked mole-rats’ gut microbiome remains stable with age, showing minimal changes in microbial populations, suggesting a robust microbiome beneficial for sustained good health. The one exception is the Archaea, Methanomassiliicoccus intestinalis, which is absent in the mouse microbiome, and increases in abundance in aging mole-rats. This comprehensive metabolomic and microbiome signature reflects evolutionary adaptations surpassing typical age-related changes, offering valuable insights into their longevity and disease resistance, with potential implications for human health interventions.

